# Adiponectin – stratification biomarker in diastolic cardiac dysfunction

**DOI:** 10.1080/14756366.2023.2171030

**Published:** 2023-01-24

**Authors:** Valeriu-Gabi Dincă, Adriana Diaconu, Bogdan-Ioan Coculescu, Alexandra-Ligia Dincă, Diana Mihaela Ciuc, Rodica Daniela Bîrlă, Cristian Daniel Marica, Sorin Ioan Tudorache, Gheorghe Manole, Elena Claudia Coculescu

**Affiliations:** a Faculty of Medicine, Titu Maiorescu University Bucharest, Bucharest, Romania; b CF2 Clinical Hospital Bucharest, Romania; c Faculty of Medicine, Carol Davila University of Medicine and Pharmacy, Bucharest, Romania; d Fundeni Clinical Institute, Bucharest, Romania; e Faculty of Midwifery and Nursing, Carol Davila University of Medicine and Pharmacy, Bucharest, Romania; f Cantacuzino National Medico-Military Institute for Research and Development, Bucharest, Romania; g Sf. Maria Clinical Hospital, Bucharest, Romania; h Faculty of General Nursing, Bioterra University, Bucharest, Romania; i Romanian Academy of Medical Sciences; j Faculty of Dental Medicine, Carol Davila University of Medicine and Pharmacy, Bucharest, Romania

**Keywords:** Adiponectin, left ventricular ejection fraction (LVEF), preserved systolic function (PRESYF), coronary artery disease (CAD), diastolic dysfunction (DD), biomarker

## Abstract

This study does not propose to elucidate how adiponectin secretion is regulated, but how its adiponectin concentration is an indicator of heart disease. About adiponectin, it is not known whether it is functionally an enzyme, or very likely a cytokine/chemokine/hormone, secreted by fat cells/adipocytes in the abdomen. Abdominal fat secretes 67 hormones, and all of which cause disease. For example, adiponectin generates diabetes and ischaemic heart disease via dyslipidemia. Based on clinical symptoms, electrocardiographic and echocardiographic parameters, a group of 208 patients with diastolic cardiac dysfunction with or without preserved systolic function, developed on a background of painful chronic ischaemic heart disease, stable angina on exertion, was constituted. The serum levels of adiponectin, total cholesterol, LDL cholesterol, HDL cholesterol and triglycerides were measured. Using the identified values, it was appreciated whether adiponectin correlates with the type of any of the two conditions, so that it can be recognised as a diagnostic and risk stratification marker.

## Introduction

Having a complex etiopathogeny, but predominantly coronary atherosclerosis, heart failure is not only one of the major causes of death, but also one of the conditions that claim high economic costs^1–4^.

Among the multifunctional hormone-type peptides, which are synthesised almost exclusively by white adipose tissue cells, there is also adiponectin, with the physiological role of regulating metabolic homeostasis and a protective effect on the cardiovascular system^5–13^. Studies on animals, as well as on humans, have shown that by reducing its plasma level, the processes induced by oxidative stress at the level of cardiomyocytes and vascular wall cells are augmented^14–18^.

Although some studies question the implication of adiponectin in atherogenesis due to slow turnover, the research results of the last two decades unanimously support that the reduction of the circulating adiponectin level as a consequence of the pathogenic mechanisms developed both at the level of the coronary wall and directly at the level of the myocardial fibre contributes etiopathogenically to the onset and evolution of diastolic cardiac dysfunction^12^^,^^19–23^.

These arguments are based on the studies of Kumada M. and Osaka from the Coronary Artery Disease Study Group, claiming that men with hypo adiponectinemia (<4.0 microg/mL) had a 2-fold increase in CAD prevalence, independent of well-known risk factors^24^. Pathogenically, it is admitted that at the level of the coronary wall, hypoadiponectinemia generates arterial dysfunction, inducing pro-inflammatory effects such as: inhibiting the activation of endothelial cells, adhesion of monocytes to endothelial cells and accelerating the transformation of macrophages into foam cells^24–27^.

At the same time, a low level of adiponectin correlates with a decrease in coronary dilatation induced by NO-synthetase activity deficiency^22^^,^^24^^,^^28–30^. Actions are the direct expression of synthesis changes in in the vascular endothelium^31–33^. In addition to the anti-atherogenic, anti-inflammatory and vasodilation actions of adiponectin, the specialised literature unanimously recognises the antioxidant, anti-apoptotic and anti-thrombotic effects^12^^,^^13^^,^^22^^,^^28^^,^^34^.

## Objectives

In the literature, it is almost unanimously accepted that the serum level of adiponectin correlates with the intensity of the protective anti-inflammatory, anti-apoptotic and vasodilation effects at arterial endothelium level. In the circumstances of a reduced serum concentration of adiponectin, the vascular pro atherogenic effects, including at the coronary level, become dominant. The induction of atherosclerosis at the level of the vessels of the heart produces, indirectly, a disturbance of the metabolic homeostasis of the myocardium, leading to the development of secondary consequences at this level. Synergistically, but directly, the reduced synthesis of adiponectin at the level of the myocardial fibre also acts to disturb the properties of the heart. In the same way, but directly, the reduced synthesis of adiponectin from myocardial fibres also acts upon the disturbance of the properties of the heart. Therefore, heart hypoadiponectinemy becomes an independent pathogenic risk factor contributing to the development of diastolic cardiac dysfunction.

The objectives aimed at in this study are:

- identifying a possible correlation between the serum concentration of LDL-cholesterol, the main serum lipoprotein involved in atherogenesis, and the level of triglycerides, on the one hand, and the level of circulating adiponectin, on the other.
- whether adiponectin level can be a disease marker, useful for risk stratification in diastolic cardiac dysfunction with or without ejection fraction reduction.

## Material and methods

The present study appoints as a prospective one following ethical principles derived from the Declaration of Helsinki (Committee on Human Investigation). The study comprises of a group of 208 patients with cardiac dysfunction on a background of 2 painful chronic ischaemic heart diseases divided into 2 subgroups, one with preserved ejection fraction (FE ≥ 50%) and another with a decrease of the ejection fraction (FE < 50%) (Table 1).

**Table 1.** Incidence of reduced serum adiponectin concentration, in the group, corresponding to the value of the ejection fraction and patients’ gender.

Types of ischaemic heart disease (target groups)	Total group							
	Regardless of gender		Gender distribution					
			Men			Women		
	No.	%	No.	%*	%**	No.	%*	%**
Group I (EF > 50%)	91	43.7	56	26.9	61.4	35	16.8	38.4
Group II (EF < 50%)	117	56.4	94	45.2	80.3	23	11	19.7
Total	208	100	150	72.1	–	58	27.8	–

In forming the group, the major concern was to ensure a good homogeneity, by excluding smokers or patients with comorbidities, such as obesity, hypertension, diabetes etc.

Since obesity is recognised as an atherogenic risk factor, both as a consequence of increased level of coexisting pro atherogenic lipid fractions, and through the secretion by adipocytes of some atherogenic hormone/adipokine peptides, we enrolled only patients with normal BMI (Body Mass Index) or with at most a deviation of plus 5% in the group^11^^,^^35–40^. Since specially designed studies have demonstrated the existence of a reduced level of adiponectin in hypertensives, only normotensive patients were enrolled^28^^,^^40^^,^^41^. Also, the study group consisted only of non-smoking patients. This is because the effect of smoking on the secretion of adiponectin has been demonstrated, as well as detected over 12 h post-smoking^42^^,^^43^. No patients with diabetes were included into the group^44–46^.

Through the elaborated investigation protocol, the patients underwent an electrocardiogram (ECG) and echocardiogram, which on the one hand allowed us to exclude from the group patients with other heart diseases, and on the other hand to evaluate the ejection fraction^47^^,^^48^.

Physiologically, knowing that plasma adiponectin levels have circadian variations, the measurement was determined from venous blood collected between 8 and 9 am, when it is assumed that adiponectin secretion reaches its maximum value. The dosage of the serum adiponectin concentration was performed via the ELISA method, from venous blood, collected in the morning, from fasting patients. Until the time of testing, although it was the same day, the collected sample was stored in a refrigerator at −30 °C.

From the collected blood samples, the lipid fractions required for the study were also determined: total cholesterol, LDL-cholesterol, HDL-cholesterol and triglycerides.

Since the circulating level of adiponectin varies according to sex, in the study, we decided to work with the average of the values determined on the groups^1^. Depending on the sex of the patients, we used their average values as normal plasma levels: 7.5 mg/L for men and 9 mg/L for women.

Regardless of gender, we considered for the entire group as a normal value of the serum adiponectin concentration the range defined by the previously mentioned average serum values (7.5–9.5 mg/L), and as an indicator of the risk existence, the limits: <7.5 mg/L = increased risk, and ≥9.5 mg/L = low risk.

## Results

a. Correlation of serum adiponectin concentration with circulating lipid fractions involved in atherogenesis.

As a function of the value of the ejection fraction, the prevalence of cases presenting serum increases in total cholesterol, its fractions and triglycerides is presented in Table 2. The number of patients with reduced serum adiponectin concentration is also presented.

**Table 2.** The prevalence of total cholesterol levels, the main circulating lipoprotein fractions, triglyceride levels and reduced serum adiponectin levels in the two groups.

The meaning of the deviation of the dosed serum factor	In the whole lot		Patients with FE > 50%		Patients with FE< 50%	
	no. abs.	%	no. abs.	%	nr. abs.	%
Total hypercholesterolaemia	172	82.1	78	37.5	94	45.1
LDL-hypercholesterolaemia	175	84.1	82	38.4	93	44.7
HDL-Hypocholesterolemia	110	52.9	49	23.5	61	29.3
Hypertriglyceridaemia	156	74.5	63	30.2	93	44.4
Reduction of serum adiponectin	157	75.5	62	29.8	95	45.6

## Discussions

Among the adipokines that act on the cardiovascular system such as tumour necrosis factor α (TNFα), leptin, plasmogen activator inhibitor-1, adipocyte fatty acid binding protein, lipocalin-2, monocyte chemotactic protein 1 and resistin, we selected adiponectin for study^19^^,^^49^^,^^50^. This peptide-hormone is secreted by both adipose tissue cells and cardiomyocytes^32^.

**Figure 1.**
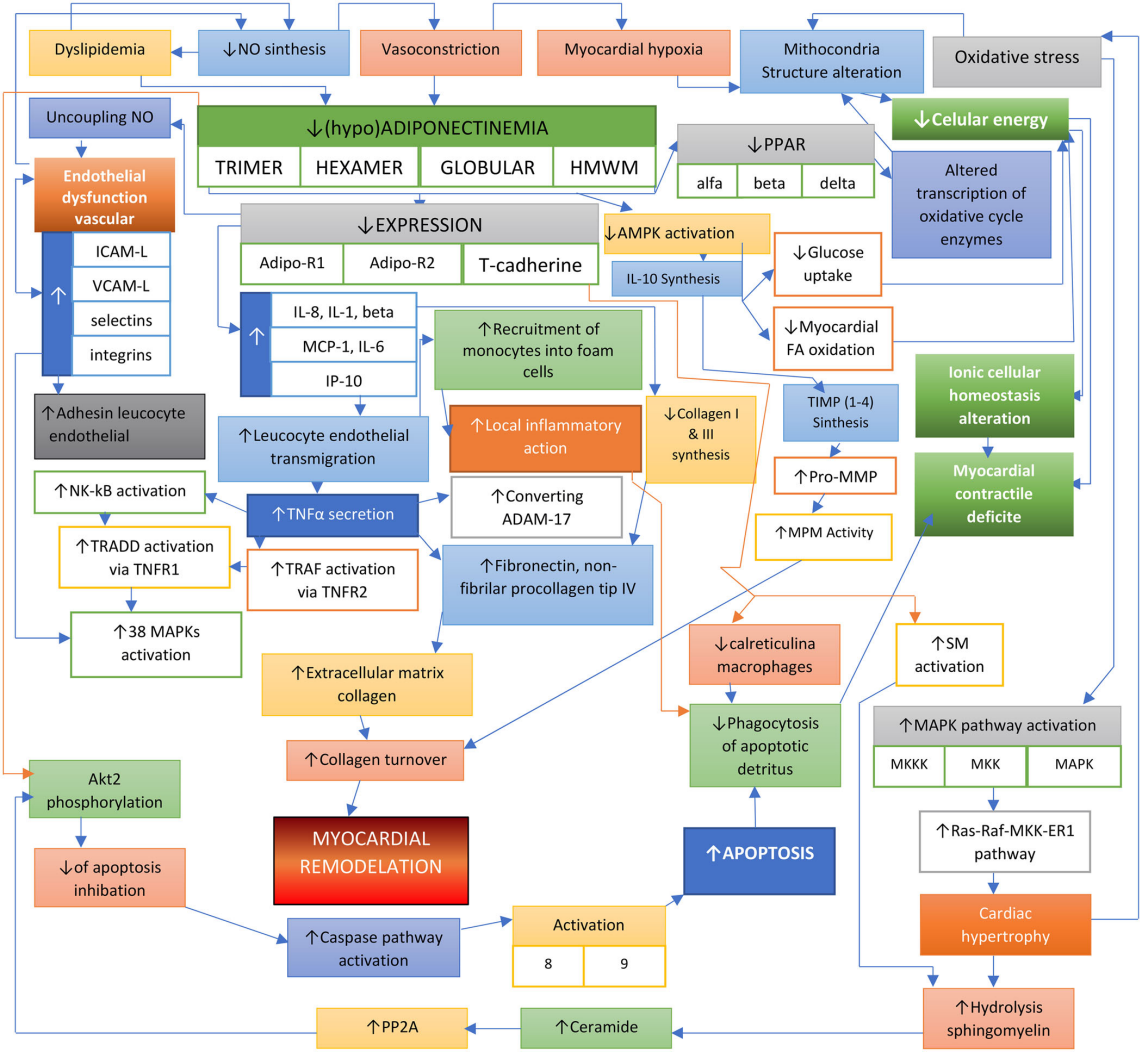
Interrelationships between reduced serum adiponectin, induced coronary artery dysfunction and dysregulation of cardiomyocyte metabolic homeostasis. FA: fatty acid; PPAR: peroxisome proliferator-activated receptor; ICAM: intercellular adhesion molecule1 (CD54); TNFR: Receptor tumour necrosis factor; NF-kB: nuclear factor kB; TIMP: inhibitors MPM-ase; AKT: proteinkinase B; VCAM-1 = vascullar cell adhesion molecular-1; ERKs(MAPKs) = MAPKs extracellular signal-regulated kinases; MAPK: mitogen-activated proteinkinases; IP10/CXCL10 = interferon ϒ-induced protein 10 kDA; MCP-1 = monocyte chemoattractant protein-1; PP2A: protein-phosphatase activators; MMP: metalaloproteinses matriceale; Ras: small GTP-ginding protein; Ras/Raf/MEK/ERK: signalling pathway; ADAM-17/TACE: disintegrin and metalloprotease 17/tumour necrosis factor-α-converting enzyme.

We justify the selection by the fact that all circulating oligomers of adiponectin [trimer, hexamer, globular and high-molecular-weight-multimer (HMWM)] at normal serum concentrations are modulators-inducers of protective effects (anti-inflammatory and anti-apoptotic) at the level of the arterial wall, but also of cardiomyocytes^11^^,^^40^^,^^51^^–^^53^ (Figure 1).

In our study we measured the serum level of both the lipoproteins involved in atherosclerosis and of adiponectin, because they can act coadjuvantically in the development of atherosclerosis (when lipid fractions such as LDL-cholesterol and triglycerides increase in the blood and the serum level of adiponectin decreases). In an aggravating sense, the hypoadiponectinemia at the myocardial level develops cardiomyopathy that adds up, pathogenically, to chronic ischaemic cardiopathy induced by atherosclerosis of the coronary vascular system. Etiopathogenically, the mechanisms contribute to disrupting the functional properties of the heart, allowing, among other conditions, the development of diastolic cardiac dysfunction.

Among the circulating lipoprotein fractions, we analysed only the possible correlation of the prevalence of LDL-hypercholesterolaemia and hypertriglyceridaemia with that of hypoadiponectinemia (Table 2).

Thus:

- In the studied group, the number of patients who presented circulating values of adiponectin below the lower limit of normal (157 patients = 75.5%) correlates in ¾ of the cases with the concomitant increase in LDL-cholesterol concentration [175 cases (84.1%) Considering that in the same group, the prevalence of hypertriglyceridaemia is 156 cases (74.5%) and this overlays on the prevalence of hypoadiponectinemia, resulting in a concordance of 99.3% (Table 2).
- The number of cases in the group with hypoadiponectinemia is identical to that of increased triglyceridemia, which allows us to draw another conclusion: the existence of a synergy of pro-atherosclerotic action of the two serum compounds.
- Much higher than the prevalence of hypertriglyceridaemia is that of cases with a high level of LDL-cholesterol (Table 2). For clinical practice, the correlation of 9 cases with hypoadiponectinemia out of 10 with LDL-hypercholesterolaemia becomes useful, indirectly indicating the way of variation of the two compounds, a way that can become useful therapeutically.
- The correlation of the prevalence of the serum concentration of hypertriglycerides with the low concentration of adiponectin is almost superposable for patients regardless of the value of the ejection fraction. However, in the case of patients with DD and reduced ejection fraction, the values are 50% higher than those with DD and PRESYF (Table 1).
- In the subgroup of patients with DD and reduced systolic flow there is a concordance of reduction in the values of systolic and serum adiponectin concentration as well. The last two analytical findings (d and e) allow us to argue that the reduction of adiponectinemia can be accepted as a risk marker, allowing the stratification.
- The data of the study highlights the dispersion of the values of the elevated circulating lipid fractions consistent with that of the reduction of the serum level of adiponectin, which allows their analysis not only individually, but also concomitantly as a complex parameter. The collective analysis increases the safety and usefulness of the information for the clinical practice of evaluating the diagnosis, but also the evolution/risk in ischaemic heart disease. This may constitute a possible multimarker in cardiovascular diseases, in particular chronic ischaemic heart disease generating diastolic cardiac dysfunction.
- In the specialised medical literature, similar studies have been carried out, but for different cardiovascular conditions, and the reported results are contradictory. It is not necessary to explain some results that do not correspond to ours, results that are from the only two published/identified articles. It is a new field of research about which nothing is known. It is known that a researcher does not take his or another’s results for granted, but puts them in contrast, as a new problem that must be studied, in depth (that is, new, more in-depth studies are needed to contribute to knowledge in the field). This is also one of our conclusions.

## Conclusions

Adiponectin has a cardiovascular protective role, both at the level of vessels and myocardium. The present paper can be interpreted as an effort to understand the mechanisms of atherosclerosis development, including the one at the coronary level and also the deterioration of the contractile function of the heart. This is because this study analyzes for the first time the evolution of serum adiponectin levels in chronic ischaemic heart disease responsible for the evolution of diastolic cardiac dysfunction.

The conclusions of the study lead to the idea of the need to carry out more studies with large number of participants, which in the eventuality of confirming our results become of real benefit to medical practice. This is because the quantification of adiponectin level in the condition of patients with dyslipidemia allows a screening of the coexistence of diastolic cardiac dysfunction, and in dynamics the peptide values allow assessment of the evolution of the disease and/or of the therapy with hypolipidemic medication.

Statistically, the degree of correlation between the increase in the serum concentration of LDL-cholesterol and triglycerides with the reduction of that of adiponectin allows us to admit that the low values of adiponectin can be a potential diagnostic biomarker and a severity indicator of diastolic dysfunction, indirectly suggesting the degree of deterioration of the ejection fraction.

## References

[1] Diaconu A, Coculescu B-I, Manole G, Vultur H, Coculescu EC, Stocheci CM, Tudorache I-S, Dincă A-L, Dincă VG. Lipoprotein-associated phospholipase A2 (Lp-PLA2) – possible diagnostic and risk biomarker in chronic ischaemic heart disease. J Enzyme Inhib Med Chem. 2021;36(1):68–73. 33187461 10.1080/14756366.2020.1839447 PMC7671608

[2] Groenewegen A, Rutten FH, Mosterd A, Hoes AW. Epidemiology of heart failure. Eur J Heart Fail. 2020;22(8):1342–1356. 32483830 10.1002/ejhf.1858 PMC7540043

[3] Snyder ML, Love S-A, Sorlie PD, Rosamond WD, Antini C, Metcalf PA, Hardy S, Suchindran CM, Shahar E, Heiss G, et al. Redistribution of heart failure as the cause of death: the atherosclerosis risk in communities study. Popul Health Metr. 2014;12(1):10. 24716810 10.1186/1478-7954-12-10 PMC4113199

[4] Velagaleti RS, Vasan RS. Heart failure in the twenty-first century: is it a coronary artery disease or hypertension problem? Cardiol Clin. 2007;25(4):487–495. 18063154 10.1016/j.ccl.2007.08.010 PMC2350191

[5] Khoramipour K, Chamari K, Hekmatikar AA, Ziyaiyan A, Taherkhani S, Elguindy NM, Bragazzi NL. Adiponectin: structure, physiological functions, role in diseases, and effects of nutrition. Nutrients. 2021;13(4):1180. 33918360 10.3390/nu13041180 PMC8066826

[6] Choi HM, Doss HM, Kim KS. Multifaceted physiological roles of adiponectin in inflammation and diseases. Int J Mol Sci. 2020;21(4):1219. 32059381 10.3390/ijms21041219 PMC7072842

[7] Liu Y, Vu V, Sweeney G. Examining the potential of developing and implementing use of adiponectin-targeted therapeutics for metabolic and cardiovascular diseases. Front Endocrinol. 2019;10:842. 10.3389/fendo.2019.00842 PMC6918867 31920962

[8] Roy B, Palaniyandi SS. Tissue-specific role and associated downstream signaling pathways of adiponectin. Cell Biosci. 2021;11(1):77. 33902691 10.1186/s13578-021-00587-4 PMC8073961

[9] Fang H, Judd RL. Adiponectin regulation and function. Compr Physiol. 2018;8(3):1031–1063. 29978896 10.1002/cphy.c170046

[10] Esfahani M, Shabab N, Saidijam M. AdipoRon may be benefit for atherosclerosis prevention. Iran J Basic Med Sci. 2017;20(2):107–109. 28293385 10.22038/ijbms.2017.8228 PMC5339649

[11] Lee B, Shao J. Adiponectin and energy homeostasis. Rev Endocr Metab Disord. 2014;15(2):149–156. 24170312 10.1007/s11154-013-9283-3 PMC4006341

[12] Lopaschuk GD, Ussher JR, Folmes CDL, Jaswal JS, Stanley WC. Myocardial fatty acid metabolism in health and disease. Physiol Rev. 2010;90(1):207–258. 20086077 10.1152/physrev.00015.2009

[13] Zhang P, Wang Y, Fan Y, Tang Z, Wang N. Overexpression of adiponectin receptors potentiates the antiinflammatory action of subeffective dose of globular adiponectin in vascular endothelial cells. Arterioscler Thromb Vasc Biol. 2009;29(1):67–74. 18988888 10.1161/ATVBAHA.108.178061

[14] Senoner T, Dichtl W. Oxidative stress in cardiovascular diseases: still a therapeutic target? Nutrients. 2019;11(9):2090. 31487802 10.3390/nu11092090 PMC6769522

[15] D’Oria R, Schipani R, Leonardini A, et al. The role of oxidative stress in cardiac disease: from physiological response to injury factor. Oxid Med Cell Longev. 2020;2020:5732956. 32509147 10.1155/2020/5732956 PMC7244977

[16] Agrimi J, Spalletti C, Baroni C, Keceli G, Zhu G, Caragnano A, Matteucci M, Chelko S, Ramirez-Correa GA, Bedja D, et al. Obese mice exposed to psychosocial stress display cardiac and hippocampal dysfunction associated with local brain-derived neurotrophic factor depletion. EBioMedicine. 2019;47:384–401. 31492565 10.1016/j.ebiom.2019.08.042 PMC6796537

[17] Kibel A, Lukinac AM, Dambic V, Juric I, Selthofer-Relatic K. Oxidative stress in ischemic heart disease. Oxid Med Cell Longev. 2020;2020:1–30. 10.1155/2020/6627144 PMC7785350 33456670

[18] Zhu C, Hu DL, Liu YQ, Zhang QJ, Chen FK, Kong XQ, Cao KJ, Zhang JS, Qian LM. Fabp3 inhibits proliferation and promotes apoptosis of embryonic myocardial cells. Cell Biochem Biophys. 2011;60(3):259–266. 21293949 10.1007/s12013-010-9148-2 PMC3112483

[19] Hui X, Lam KSL, Vanhoutte PM, Xu A. Adiponectin and cardiovascular health: an update. Br J Pharmacol. 2012;165(3):574–590. 21457225 10.1111/j.1476-5381.2011.01395.x PMC3315032

[20] López-Jaramillo P, Gómez-Arbeláez D, López-López J, López-López C, Martínez-Ortega J, Gómez-Rodríguez A, Triana-Cubillos S. The role of leptin/adiponectin ratio in metabolic syndrome and diabetes. Horm Mol Biol Clin Investig. 2014;18(1):37–45. 10.1515/hmbci-2013-0053 25389999

[21] Wang ZV, Scherer PE. Adiponectin, the past two decades. J Mol Cell Biol. 2016;8(2):93–100. 26993047 10.1093/jmcb/mjw011 PMC4816148

[22] Lau WB, Ohashi K, Wang Y, Ogawa H, Murohara T, Ma X-L, Ouchi N. Role of adipokines in cardiovascular disease. Circ J. 2017;81(7):920–928. 28603178 10.1253/circj.CJ-17-0458

[23] Parker-Duffen JL, Walsh K. Cardiometabolic effects of adiponectin. Best Pract Res Clin Endocrinol Metab. 2014;28(1):81–91. 24417948 10.1016/j.beem.2013.09.001 PMC3905311

[24] Kumada M, Kihara S, Sumitsuji S, Kawamoto T, Matsumoto S, Ouchi N, Arita Y, Okamoto Y, Shimomura I, Hiraoka H, et al. Study Group. Coronary artery disease. Association of hypoadiponectinemia with coronary artery disease in men. Arterioscler Thromb Vasc Biol. 2003;23(1):85–89. 12524229 10.1161/01.atv.0000048856.22331.50

[25] Theofilis P, Sagris M, Oikonomou E, Antonopoulos AS, Siasos G, Tsioufis C, Tousoulis D. Inflammatory mechanisms contributing to endothelial dysfunction. Biomedicines. 2021;9(7):781. 34356845 10.3390/biomedicines9070781 PMC8301477

[26] Coculescu BI, Manole G, Coculescu EC, et al. Autophagy as a neuronal survival mechanism in ischemic stroke. Rom J Leg Med. 2018;26(4):333–339.

[27] Lin P, Ji H-H, Li Y-J, Guo S-D. Macrophage plasticity and atherosclerosis therapy. Front Mol Biosci. 2021;8:679797. 34026849 10.3389/fmolb.2021.679797 PMC8138136

[28] Ebrahimi-Mamaeghani M, Mohammadi S, Arefhosseini SR, Fallah P, Bazi Z. Adiponectin as a potential biomarker of vascular disease. Vasc Health Risk Manag. 2015;11:55–70. 25653535 10.2147/VHRM.S48753 PMC4303398

[29] Ouchi N, Walsh K. Adiponectin as an anti-inflammatory factor. Clin Chim Acta. 2007;380(1–2):24–30. 17343838 10.1016/j.cca.2007.01.026 PMC2755046

[30] Fisman EZ, Tenenbaum A. Adiponectin: a manifold therapeutic target for metabolic syndrome, diabetes, and coronary disease? Cardiovasc Diabetol. 2014;13:103. 24957699 10.1186/1475-2840-13-103 PMC4230016

[31] da Silva Rosa SC, Liu M, Sweeney G. Adiponectin synthesis, secretion and extravasation from circulation to interstitial space. Physiology. 2021;36(3):134–149. 33904786 10.1152/physiol.00031.2020 PMC8461789

[32] Aljafary MA, Al-Suhaimi EA. Adiponectin system (Rescue Hormone): the missing link between metabolic and cardiovascular diseases. Pharmaceutics. 2022;14(7):1430. 35890325 10.3390/pharmaceutics14071430 PMC9321059

[33] (a) Cohen KE, Katunaric B, SenthilKumar G, McIntosh JJ, Freed JK. Vascular endothelial adiponectin signaling across the life span. Am J Physiol Heart Circ Physiol. 2022;322(1):H57–H65. 34797171 10.1152/ajpheart.00533.2021 PMC8698498

[34] Nigro E, Scudiero O, Monaco ML, Palmieri A, Mazzarella G, Costagliola C, Bianco A, Daniele A. New insight into adiponectin role in obesity and obesity-related diseases. Biomed Res Int. 2014;2014:658913. 25110685 10.1155/2014/658913 PMC4109424

[35] Caselli C, D’Amico A, Cabiati M, Prescimone T, Del Ry S, Giannessi D. Back to the heart: the protective role of adiponectin. Pharmacol Res. 2014;82:9–20. 24657240 10.1016/j.phrs.2014.03.003

[36] Yoo HJ, Choi KM. Adipokines as a novel link between obesity and atherosclerosis. World J Diabetes. 2014;5(3):357–363. 24936256 10.4239/wjd.v5.i3.357 PMC4058739

[37] Zorena K, Jachimowicz-Duda O, Ślęzak D, Robakowska M, Mrugacz M. Adipokines and obesity. Potential link to metabolic disorders and chronic complications. Int J Mol Sci. 2020;21(10):3570. 32443588 10.3390/ijms21103570 PMC7278967

[38] Landecho MF, Tuero C, Valentí V, Bilbao I, de la Higuera M, Frühbeck G. Relevance of leptin and other adipokines in obesity-associated cardiovascular risk. Nutrients. 2019;11(11):2664. 31694146 10.3390/nu11112664 PMC6893824

[39] Kim DH, Kim C, Ding EL, Townsend MK, Lipsitz LA. Adiponectin levels and the risk of hypertension: a systematic review and meta-analysis. Hypertension. 2013;62(1):27–32. 23716587 10.1161/HYPERTENSIONAHA.113.01453 PMC3729220

[40] Zhuang L, Li C, Chen Q, Jin Q, Wu L, Lu L, Yan X, Chen K. Fatty acid-binding protein 3 contributes to ischemic heart injury by regulating cardiac myocyte apoptosis and MAPK pathways. Am J Physiol Heart Circ Physiol. 2019;316(5):H971–H984. 30735072 10.1152/ajpheart.00360.2018

[41] Matsuda M, Shimomura I. Roles of adiponectin and oxidative stress in obesity-associated metabolic and cardiovascular diseases. Rev Endocr Metab Disord. 2014;15(1):1–10. 24026768 10.1007/s11154-013-9271-7

[42] Kryfti M, Dimakou K, Toumbis M, Daniil Z, Hatzoglou C, Gourgoulianis KI. Effects of smoking cessation on serum leptin and adiponectin levels. Tob Induc Dis. 2015;13:30. 26869871 10.1186/s12971-015-0054-7 PMC4750367

[43] Iwashima Y, Katsuya T, Ishikawa K, Kida I, Ohishi M, Horio T, Ouchi N, Ohashi K, Kihara S, Funahashi T, et al. Association of hypoadiponectinemia with smoking habit in men. Hypertension. 2005;45(6):1094–1100. 15897361 10.1161/01.HYP.0000169444.05588.4c

[44] Mohammadi S, Hasseinzadeh Attar MJ, Karimi M, et al. The effects of green tea extract on serum adiponectin concentration and insulin resistance in patients with Type 2 Diabetes Mellitus. J Adv Med Biomed Res. 2010;18(70):44–57.

[45] Anton IC, Botnariu EG, Coculescu EC, Boanca M, Mititelu-Tartau LM. Particular aspects of evolution of SARS-Cov-2 infection in type 2 diabetic patients. Rom J Leg Med. 2021;29(1):53–59.

[46] Gîrlescu N, Botnariu EG, Coculescu EC, Hunea I, Diac M, Gheorghe L, Bulgaru Iliescu D. The haze of diabetic state in legal medicine – a review. Rom J Leg Med. 2021;29(2):212–217.

[47] Olesen LL, Andersen A. ECG as a first step in the detection of left ventricular systolic dysfunction in the elderly. ESC Heart Fail. 2016;3(1):44–52. 27774266 10.1002/ehf2.12067 PMC5061087

[48] Lenarduzzi Júnior RM, de Almeida Neto OP, Pedrosa LA, Silva PC, Coelho VM, Resende ES, Mendes DS. Electrocardiographic and echocardiographic profile of patients with heart failure. Am J Cardiovasc Dis. 2021;11(6):695–703. 35116181 PMC8784673

[49] Jang D-i, Lee A-H, Shin H-Y, Song H-R, Park J-H, Kang T-B, Lee S-R, Yang S-H. The role of tumor necrosis factor Alpha (TNF-α) in autoimmune disease and current TNF-α inhibitors in therapeutics. Int J Mol Sci. 2021;22(5):2719. 33800290 10.3390/ijms22052719 PMC7962638

[50] Oh DK, Ciaraldi T, Henry RR. Adiponectin in health and disease. Diabetes Obes Metab. 2007;9(3):282–289. 17391153 10.1111/j.1463-1326.2006.00610.x

[51] Jung HN, Jung CH. The role of anti-inflammatory adipokines in cardiometabolic disorders: moving beyond adiponectin. Int J Mol Sci. 2021;22(24):13529. 34948320 10.3390/ijms222413529 PMC8707770

[52] Robinson K, Prins J, Venkatesh B. Clinical review: adiponectin biology and its role in inflammation and critical illness. Crit Care. 2011;15(2):221. 21586104 10.1186/cc10021 PMC3219307

[53] Song HJ, Oh S, Quan S, Ryu O-H, Jeong J-Y, Hong K-S, Kim D-H. Gender differences in adiponectin levels and body composition in older adults: Hallym aging study. BMC Geriatr. 2014;14:8. 24460637 10.1186/1471-2318-14-8 PMC3931323

